# Type 2 Diabetic Mellitus Is a Risk Factor for Nasopharyngeal Carcinoma: A 1:2 Matched Case–Control Study

**DOI:** 10.1371/journal.pone.0165131

**Published:** 2016-10-19

**Authors:** Xing-Si Peng, Guo-Feng Xie, Wen-Ze Qiu, Yun-Hong Tian, Wei-Jun Zhang, Ka-Jia Cao

**Affiliations:** 1 Department of Nasopharyngeal Carcinoma, Sun Yat-sen University Cancer Center, Guangzhou, Guangdong, China; 2 State Key Laboratory of Oncology in South China, Sun Yat-sen University Cancer Center, Guangzhou, Guangdong, China; 3 Collaborative Innovation Center of Cancer Medicine, Sun Yat-sen University Cancer Center, Guangzhou, Guangdong, China; 4 Department of Radiation Oncology, Cancer Center of Guangzhou Medical University, Guangzhou, Guangdong, China; Chang Gung University, TAIWAN

## Abstract

**Background:**

Diabetes has been identified as an adverse prognostic variable which associated with an increased mortality in various cancers, including colorectal, lung, and breast cancers. However, previous studies provided inconsistent results on the association between diabetes and nasopharyngeal carcinoma (NPC). The main aim of this study was to investigate the associations between diabetes mellitus and the survival of NPC patients.

**Methods:**

This study was designed as a 1:2 matched case–control study. Cases were patients who met the criteria for the diagnosis of type 2 diabetic mellitus (DM) below. Controls, matched 1:2, were patients who were normoglycemic (NDM). The survival rates were assessed by Kaplan–Meier analysis, and the survival curves were compared using a log-rank test. Multivariate analysis was conducted using the Cox proportional hazard regression model.

**Results:**

Both locoregional relapse-free survival (LRRFS) and disease-free survival (DFS) in the NDM group were higher than that in the DM group (p = 0.001 and p = 0.033). Additionally, subset analyses revealed that the differences in OS, LRRFS, and DFS were all significant between the two groups in the N0-N1 subset (p = 0.007, p =.000 and p = 0.002). The LRRFS was higher in the NDM group in the III-IV, T3-T4 and N0-N1 subsets (p = 0.004, p = 0.002 and p =.000). In T3-T4 subset, the NDM group experienced higher DFS than the DM group (p = 0.039). In multivariate analysis, T stage and N stage were found to be independent predictors for OS, DMFS and DFS; chemotherapy was a significant prognostic factor for DMFS and DFS, age for OS, and diabetes for LRRFS and DFS.

**Conclusions:**

Type 2 diabetic mellitus is associated with poorer prognosis among patients with NPC.

## Introduction

Type 2 diabetes mellitus (hereafter referred to as diabetes) is increasing rapidly worldwide. Epidemiological studies suggest that individuals with diabetes mellitus are at higher risk of cancer [1]. Moreover, diabetes has been identified as an adverse prognostic variable associated with increased mortality in various cancers, including colorectal cancer [2], lung cancer [3], and breast cancer [4].

To date, there were only three studies about diabetes and the prognosis of NPC. Previous studies provided inconsistent results on the association between diabetes and NPC. In a study by Liu et al [5], DFS in patients with diabetes was poorer than in those without diabetes. While OuYang et al [6] and Hao Peng et al [7] found that diabetic and prediabetic NPC patients had similar survival to normoglycemic NPC patients. All these previous studies were cohort studies, which may not get more reliable results inevitably caused by confounding factors like gender, age, T stage, N stage, clinical stage, radiotherapy, chemotherapy.

In this first case-control study taken by multi-center departments with large sample size, the main aim was to investigate the associations between diabetes mellitus and the survival of NPC patients.

## Methods and Materials

This study was designed as a 1:2 matched case–control study.

### Patient selection

The study was approved by the Research Ethic Committee of Sun Yat-sen University Cancer Center (YB2015-042-01) and Cancer Center of Guangzhou Medical University (2016–81) and written informed consent was obtained from each patient.

We retrospectively analyzed data from 4236 hospitalized patients diagnosed with NPC between November 2007 and January 2011 at Sun Yat-sen University Cancer Center, and data from 4062 hospitalized patients diagnosed with NPC between November 2003 and January 2011 at Cancer Center of Guangzhou Medical University. NPC patients were pathologically diagnosed with non-keratinizing or undifferentiated carcinoma of the nasopharynx (World Health Organization [WHO] type II or III), without distant metastasis. All the NPC patients had completed radical radiotherapy, and patients who had important organ dysfunction or other uncontrolled serious diseases, and those who received previously other treatments for NPC were excluded. Cases were patients who met the criteria for the diagnosis of DM below without complications. Controls, matched 1:2, were patients who were NDM. An eligible control was matched to a case by gender, age (within 5 years), T stage, N stage, chemotherapy (with or not) and radiotherapy (2-dimentional radiotherapy or IMRT). There were 186 patients included in DM group, and 372 in NDM group.

### Diagnosis of DM

Diagnosis of type 2 diabetic mellitus was based upon the 2012 American Diabetes Association (ADA) guidelines. According to these guidelines, patients must meet any of the following: (1) symptoms of diabetes at any time + plasma glucose ≥11.1mmol / L; (2) fasting plasma glucose ≥7mmol / L; (3) 2-hour postprandial blood glucose ≥11.1mmol / L.

### Clinical staging

All the methods in this current study were carried out in accordance with the approved guidelines [8]. The routine staging process included a complete medical history and clinical examination of the head and neck region, direct fiber-optic nasopharyngoscopy, magnetic resonance imaging (MRI) of the skull base and the entire neck, chest radiography, a whole-body bone scan, abdominal sonography and positron emission tomography (PET)-CT. Tumor-associated markers immunoglobulin A (IgA) antibodies to EBV viral capsid antigen (VCA) and to EBV early antigen (EA) were tested, along with plasma EBV DNA. All patients had a dental evaluation before radiotherapy and were restaged according to the 2002 Union for International Cancer Control (UICC) Staging System. All MRI materials and clinical records were reviewed to minimize heterogeneity in restaging.

### Treatment

#### Radiotherapy

All patients were treated with 2-dimentional radiotherapy or IMRT at Sun Yat-sen University Cancer Center and Cancer Center of Guangzhou Medical University. The prescribed doses were 66–72 Gy/ 30–36 fraction to the planning target volume (PTV) of the primary gross tumor volume (GTVnx), and 60–78 Gy/ 30–34 fraction to the PTV of the GTV of the involved lymph nodes (GTVnd).

#### Chemotherapy

Before all treatment, we recommended radiotherapy alone for stage I patients, concurrent chemoradiotherapy for stage II patients, and concurrent chemoradiotherapy (CCRT) +/− neoadjuvant/ adjuvant chemotherapy for stage III to IV patients, according to our institutional guidelines. Neoadjuvant chemotherapy was given mainly when the waiting time was unacceptably long or when it was considered advantageous to reduce the size of large tumors. Neoadjuvant or adjuvant chemotherapy consisted of cisplatin with 5- uorouracil, cisplatin with taxoids or cisplatin with both 5-uorouracil and toxoids, applied every three weeks for two or three cycles. Concurrent chemotherapy consisted of cisplatin given weekly or on weeks 1, 4 and 7 of radiotherapy.

### Statistical analysis

We used the SPSS 22.0 statistical software (SPSS Inc., Chicago, IL, USA). We used Chi square analysis to compare occurrence rates of adverse events and categorical variables. Patient death, relapse of a local or nodal tumor, occurrence of distant metastasis, and occurrence of relapse or distant metastasis respectively determined the study end-points of OS, LRRFS, DMFS, and DFS. The Kaplan–Meier method was used to calculate the time-to-event for each endpoint from the date of start of treatment to the occurrence date of the event. Statistical differences in the endpoints were estimated using the log-rank test. The multivariate analysis was conducted by the Cox proportional hazard regression model. A two-tailed *P* value of less than 0.05 was considered significant.

## Results

### Patient characteristics

Between January 2008 and December 2010, clinical data of 288 NPC patients treated in the Sun Yat-sen University Cancer Center and 270 NPC patients treated in the Cancer Center of Guangzhou Medical University who met all of the criteria of the matched case control study were retrospectively analyzed. The clinical characteristics of the 558 NPC patients are listed in Table 1. Of these patients, 480 were males and 78 were females. The median age was 53 years. Of the 558 patients, 186/558 (33.3%) were diagnosed with DM, and 372/558 (66.7%) were NDM. No significant differences were found between the two groups in baseline characteristics (Table 1).

**Table 1:** Characteristics of the 558 patients with NPC.

Characteristic	NDM [cases (%)]	DM [cases (%)]	*X*^*2*^	*P*
**Total**	372 (66.7)	186(33.3)		
**Age (years)**			1.642	0.200
**<50**	157 (42.2)	68 (36.6)		
**≥50**	215 (57.8)	118 (63.4)		
**Gender**			.000	1.000
**Male**	320 (86.0)	160 (86.0)		
**Female**	52 (14.0)	26 (14.0)		
**T stage**			0.066	0.996
**T1**	24 (6.5)	13 (7.0)		
**T2**	114 (30.6)	56 (30.1)		
**T3**	158 (42.5)	79 (42.5)		
**T4**	76 (20.4)	38 (20.4)		
**N stage**			0.024	0.999
**N0**	96 (25.8)	48 (25.8)		
**N1**	96 (25.8)	47 (25.3)		
**N2**	128 (34.4)	65 (34.9)		
**N3**	52 (14.0)	26 (14.0)		
**Overall stage**			.000	1.000
**I**	4 (1.1)	2 (1.1)		
**II**	78 (21.0)	39 (21.0)		
**III**	188 (50.5)	94 (50.5)		
**IV**	102 (27.4)	51 (27.4)		
**Chemotherapy**			0.005	0.946
**Yes**	275 (73.9)	138 (74.2)		
**No**	97 (26.1)	48 (25.8)		
**Radiotherapy**			0.018	0.894
**2DRT**	106 (28.5)	52 (28.0)		
**IMRT**	266 (71.5)	134 (72.0)		

DM = type 2 diabetic mellitus, NDM = normoglycemic, 2DRT = 2-dimentional radiotherapy, IMRT = intensity-modulated radiotherapy.

### Treatment and compliance

All patients completed the full course of radiotherapy. 158/558 (28.3%) were treated with 2-Dimensional Radiation Therapy (2DRT), and 400/558 (71.7%) were treated with Intensity- Modulated Radiation Therapy (IMRT). Of all these patients, 413/558 (74.0%) received chemotherapy and 145/558 (26%) did not. The 186 NPC patients diagnosed with DM received insulin injection or oral antidiabetic drugs during the full course of radiotherapy and chemotherapy.

### Failure patterns

The median follow-up time was 66 months (range, 3–157 months), and 9 (1.6%) patients were lost to follow-up. Treatment failure patterns are summarized in Table 2. DM patients experienced higher locoregional failure than NDM patients (16.1% vs. 7.5%; p = 0.02), higher rates of combined locoregional plus distant failure (3.2% vs. 0.5%; p = 0.012), and also experienced higher rates of locoregional or distant failure (28.0% vs. 20.0%; p = 0.038) (Table 2).

**Table 2:** Patterns of disease failure in the two groups of NDM and DM.

Failure pattern	NDM [cases (%)]	DM [cases (%)]	*P*
**Locoregional relapse**	28 (7.5)	30 (16.1)	**0.002**
**Distant metastasis**	49 (13.2)	28 (15.1)	0.543
**Both locoregional relapse and distant metastasis**	2 (0.5)	6 (3.2)	**0.012**
**Locoregional relapse or distant metastasis**	75 (20.2)	52 (28.0)	**0.038**
**Death**	90 (24.2)	52 (28.0)	0.336

DM = type 2 diabetic mellitus, NDM = normoglycemic.

### Survival analysis

**Fig 1:**
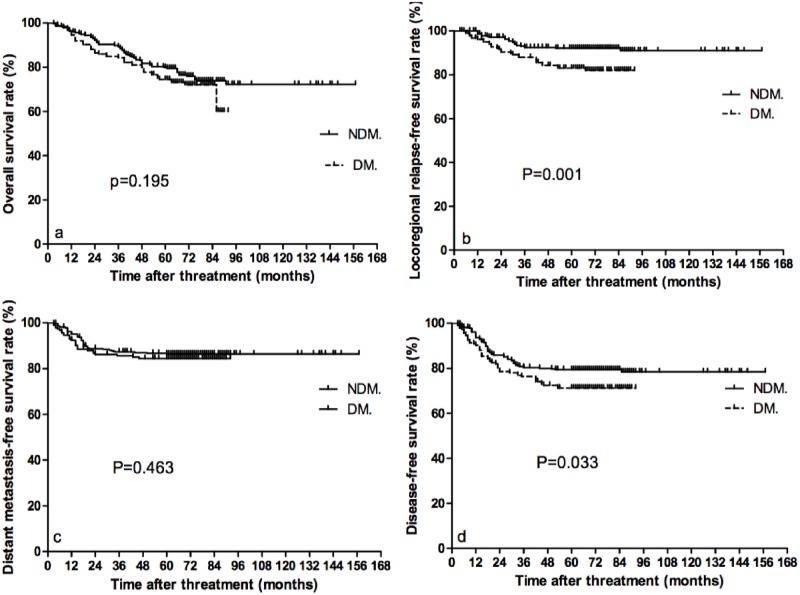
Kaplan–Meier estimates of the survival of the two groups in NDM and DM. (a) overall survival; (b) locoregional relapse-free survival; (c) distant metastasis-free survival; (d) disease-free survival

The 5-year OS, LRRFS, DMFS, and DFS rates were 80.1, 92.1, 86.3, and 79.5%, respectively, for the NDM group, and 74.5, 83.0, 84.4, and 71.3%, respectively, for the DM group. There were significant differences in both LRRFS and DFS between the two groups (Fig 1). Both LRRFS and DFS in the NDM group were higher than that in the DM group (p = 0.001 and p = 0.033). Additionally, subset analyses revealed that the differences in OS, LRRFS, and DFS were all significant between the two groups in the N0-N1 subset (p = 0.007, p =.000 and p = 0.002). The LRRFS was higher in the NDM group in the III-IV, T3-T4 and N0-N1 subsets (p = 0.004, p = 0.002 and p =.000). Of T3-T4 patients, the NDM group experienced higher DFS than the DM group (p = 0.039) (Table 3).

**Table 3:** Subset analyses on tumor control in the two groups of NDM and DM.

Stage	OS		LRRFS		DMFS		DFS	
	HR (95%CI)	*P*	HR (95%CI)	*P*	HR (95%CI)	*P*	HR (95%CI)	*P*
**Overall**	1.25 (0.89–1.77)	0.196	2.30 (1.37–3.85)	**0.002**	1.19 (0.75–1.89)	0.466	1.47 (1.03–2.09)	**0.034**
**I-II**	2.53 (0.75–8.56)	0.137	2.26 (0.57–9.06)	0.249	4.14 (0.38–45.64)	0.246	2.65 (0.81–8.69)	0.108
**III-IV**	1.18 (0.82–1.69)	0.373	2.26 (1.30–3.94)	**0.004**	1.12 (0.69–1.80)	0.645	1.37 (0.94–1.98)	0.100
**T1-T2**	1.15 (0.51–2.63)	0.737	1.69 (0.63–4.55)	0.301	0.76 (0.20–2.87)	0.687	1.31 (0.60–2.90)	0.500
**T3-T4**	1.30 (0.89–1.89)	0.176	0.26 (1.41–4.76)	**0.002**	1.27 (0.77–2.09)	0.343	1.52 (1.02–2.25)	**0.039**
**N0-N1**	2.21 (1.24–3.94)	**0.007**	4.48 (2.01–9.99)	**0.000**	1.76 (0.76–4.08)	0.185	2.54 (1.41–4.56)	**0.002**
**N2-N3**	0.93 (0.60–1.44)	0.750	1.29 (0.62–2.65)	0.495	0.99 (0.57–1.74)	0.980	1.05 (0.67–1.66)	0.835

HR = hazard ratio, CI = confidence interval, OS = overall survival, LRRFS = loco-regional relapse-free survival, DMFS = distant metastasis-free survival, DFS = disease-free survival.

### Prognostic factors

The various potential prognostic factors for predicting LRRFS, DMFS, DFS, and OS rates, including gender, age, T stage, N stage, overall stage, chemotherapy, radiotherapy and diabetes, were evaluated in univariate and multivariate analysis. In univariate analysis, T stage, N stage, overall stage and chemotherapy were significant prognostic factors for OS, DMFS, and DFS (Table 4). In multivariate analysis, T stage and N stage were found to be independent predictors for OS, DMFS and DFS; chemotherapy was a significant prognostic factor for DMFS and DFS, age for OS, and diabetes for LRRFS and DFS (Table 5).

**Table 4:** Univariate analysis of prognostic factors for the two groups of NPC patients.

Variate	5-year survival rate (%)							
	OS	*P*	LRRFS	*P*	DMFS	*P*	DFS	*P*
**Gender**		0.167		0.913		0.963		0.742
Male	78.9		89.2		85.9		76.9	
Female	74.4		88.5		86.0		75.5	
**Age (years)**		**0.018**		0.146		0.452		0.115
<50	83.1		91.1		87.0		79.9	
≥50	74.9		87.7		85.1		74.6	
**T stage**		**.000**		0.065		**.000**		**.000**
T1-2	91.8		92.4		95.1		87.8	
T3-4	70.3		87.0		80.2		70.0	
**N stage**		**.000**		0.188		**.000**		**.000**
N0-1	86.4		91.0		92.2		84.5	
N2-3	69.5		86.9		78.9		68.3	
**Overall stage**		**.000**		0.063		**.000**		**.000**
I-II	95.1		94.2		97.6		91.8	
III-IV	73.4		87.6		82.5		72.4	
**Chemotherapy**		**.000**		0.064		**.000**		**.000**
No	89.7		93.6		97.9		92.2	
Yes	74.2		87.4		81.7		71.3	
**Radiotherapy**		0.993		0.300		0.831		0.775
2DRT	80.2		87.6		86.2		77.7	
IMRT	77.4		89.7		85.8		76.4	
**Diabetes**		0.195		**0.001**		0.465		**0.033**
NDM	80.1		92.1		86.3		79.5	
DM	74.5		83.0		84.4		71.3	

OS = overall survival, LRRFS = loco-regional relapse-free survival, DMFS = distant metastasis-free survival, DFS = disease-free survival, 2DRT = 2-dimentional radiotherapy, IMRT = intensity-modulated radiotherapy, DM = type 2 diabetic mellitus, NDM = normoglycemic.

**Table 5:** Multivariate analysis of prognostic factors for the two groups of NPC patients.

Variable	OS		LRRFS		DMFS		DFS	
	HR (95%CI)	*P*	HR (95%CI)	*P*	HR (95%CI)	*P*	HR (95%CI)	*P*
**Gender (males vs. females)**	0.80 (0.51–1.25)	0.331	1.01 (0.47–2.16)	0.980	1.19 (0.61–2.33)	0.609	1.06 (0.64–1.75)	0.827
**Age (<50 vs. ≥50 years)**	1.57 (1.10–2.24)	**0.013**	1.41 (0.81–2.46)	0.221	1.22 (0.76–1.94)	0.414	1.31 (0.91–1.89)	0.152
**T stage (T1-2 vs. T3-4)**	3.10 (1.74–5.52)	**.000**	1.45 (0.64–3.29)	0.369	2.76 (1.30–5.88)	**0.008**	2.20 (1.25–3.88)	**0.006**
**N stage (N0-1 vs. N2-3)**	2.37 (1.57–3.59)	**.000**	1.27 (0.67–2.39)	0.461	2.13 (1.23–3.69)	**0.007**	1.85 (1.21–2.84)	**0.005**
**Overall stage (I-II vs. III-IV)**	0.73 (0.30–1.78)	0.482	1.03 (0.32–3.34)	0.960	1.06 (0.25–4.53)	0.942	0.82 (0.33–2.02)	0.667
**Chemotherapy (No vs. Yes)**	1.33 (0.75–2.37)	0.331	1.53 (0.69–3.40)	0.296	4.70 (1.38–16.02)	**0.013**	2.32 (1.18–4.54)	**0.014**
**Radiotherapy (2DRT vs. IMRT)**	0.81 (0.56–1.17)	0.257	0.65 (0.37–1.14)	0.135	0.81 (0.49–1.35)	0.420	0.85 (0.57–1.26)	0.422
**Diabetes (NDM vs. DM)**	1.23 (0.87–1.74)	0.235	2.27 (1.35–3.80)	**0.002**	1.18 (0.74–1.88)	0.496	1.44 (1.01–2.05)	**0.046**

HR = hazard ratio, CI = confidence interval, OS = overall survival, LRRFS = loco-regional relapse-free survival, DMFS = distant metastasis-free survival, DFS = disease-free survival, 2DRT = 2-dimentional radiotherapy, IMRT = intensity-modulated radiotherapy, DM = type 2 diabetic mellitus, NDM = normoglycemic.

## Discussion

As far as we know, this is the first report of a matched case-control study and taken by two of the largest cancer centers in South China to investigate the impact of diabetes on the prognosis of non-metastatic NPC patients. Our results indicate that DM is an independent prognostic factor for LRRFS and DFS in non-metastatic NPC patients treated after radiotherapy and chemotherapy. These results have particular impact for populations in South China where NPC is prevalent.

To date, only three studies have examined cancer-specific mortality among patients with NPC with or without diabetes. According to OuYang et al [6], the diabetic and prediabetic NPC patients had similar survival to normoglycemic NPC patients. Recently, Hao Peng et al [7] also found that diabetes had no prognostic impact on NPC patients treated using IMRT. However, in the study of Liu et al [5], the diabetes group had a worse DFS than the non-diabetes group. All of the three previous researches were retrospective cohort studies. The inconsistent results of these studies may be due to the difference on the sample sizes (N = 37, 81 and 345) and the confounding factors caused by cohort studies. Many factors, including sex, age, T stage, N stage, clinical stage, radiotherapy, chemotherapy and more can affect OS, DFS, MFS, and RFS of patients with NPC [9]. To reduce the impact of these confounding factors, a matched case-control design from a large cohort of NPC patients is considered to be a more objective method to draw a more reliable conclusion, In our study, the results show that NPC patients with DM have a poorer 5-year LRRFS and DFS than non-diabetic patients. Additionally, subset analyses revealed that for N0-N1 NPC patients, DM was an independent prognostic factor in OS, LRRFS and DFS. Overall, our results suggest that for NPC patients with type 2 diabetes, special measures may be needed [10, 11] to improve the local control rate and to also reduce tumor recurrence rate.

At present, many studies have found that diabetes not only increases the incidence of breast cancer [12], liver cancer [13], and lung cancer [14], but also affects the prognosis of pancreatic cancer [15, 16], colon cancer [17, 18] and other cancers [19, 20], which are in agreement with our study. There are several potential explanations for the observed association between DM and higher risk of mortality in patients with cancers such as colorectal [21–23] and breast [24, 25] cancers. Hyperglycemia promotes tumor growth and neoplastic proliferation [26]. It is known that insulin resistance, hyperinsulinaemia and elevated levels of IGF-1 promote tumor cell growth [27]. High insulin and insulin-like growth factor (IGF) may stimulate insulin-mediated mutagenesis and cancer cell proliferation and metastasis [28]. Moreover, diabetes could influence NPC survival through alternative mechanisms. Mortality from such hyperglycemia-related complications as hypertension, heart diseases and various hyperlipidemias may reduce differences in DFS, MFS and RFS if patients with diabetes died before recurrence or metastasis. Dehal et al [29] found that patients with colorectal cancer and DM exhibit an especially higher risk of death from cardiovascular disease. In this retrospective study, the accurate information about the causes of non-cancer death was unavailable, so we need to do more further fundamental and clinical researches to make clear of the mechanism between diabetes and NPC.

It was a retrospective study, detail information regarding the subclassification and stage of diabetes, the treatment on diabetes and the changing levels of blood sugar, were not available for most patients. Therefore, multi-center studies with large samples or prospective case-control studies are needed in the future. We recommend the collection of more clinical, pathological, and treatment data to determine the effect of DM on prognosis of NPC.

In conclusion, this study supports the hypothesis that DM is associated with poorer prognosis among patients with NPC. What is more, NPC patients with DM experience poorer LRRFS and DFS.

## Supporting Information

S1 File Data set for SPSS analysis. Note: All the results in this study were drawn from this data set.
(SAV)

S2 File Numbers from IRB. (ZIP)
