# Supplementary material for: Type 2 Diabetic Mellitus Is a Risk Factor for Nasopharyngeal Carcinoma: A 1:2 Matched Case–Control Study
Source: PLoS One. 2016 Oct 19;11(10):e0165131. doi: 10.1371/journal.pone.0165131 (PMC5070777; doi:10.1371/journal.pone.0165131)
Supplement: S2 File — (ZIP) [file pone.0165131.s002.zip › number from IRB.pdf]

Sun Yat-sen University Cancer Center IRB

中山大学肿瘤防治中心伦理委员会

审批号/ **Approval No**：YB2015-042-01　　审批日期/ **Approval Date**：Oct. 12, 2015

地址：广州市越秀区东风东路 651 号　邮编：510060　电话/Tel：87343135　传真/Fax：87343009

Address：651，Dongfeng East Road，　Guangzhou, China 510060

Protocol title：Type 2 diabetic mellitus is a risk factor for nasopharyngeal carcinoma: a 1:2 matched case–control study

Protocol version & Date: 01 & September 1,2015

Informed consent version & Date: Exempted.

Study site & Principle Investigator: Sun Yat-sen University Cancer Center & Ka-Jia, Cao

Two IRB members of SYSUCC have expedited review the proposal on Oct. 12, 2015 in accordance to ICH GCP guidelines, government regulations and laws, and agreed to approve this protocol.

Conditions: 1. Do not deviate from, or make changes to the study protocol without prior written IRB approval, except when it is necessary to eliminate immediate hazards to research subjects or when the change involves only logistical or administrative issues; 2. Report the following to SYSUCC IRB: (1) study protocol or consent document change, (2) serious adverse event, (3) study progress, and (4) new information that may be relevant to a subject's willingness to continue participation in the study; 3. Report study progress to SYSUC IRB at a 12-monthly interval until study closure.

Peng Wang qing

Peng Wang-qing

IRB Chairman

Sun Yat-sen University Cancer Center

2015-10-12
