# Supplementary figures and images for: Type 2 Diabetic Mellitus Is a Risk Factor for Nasopharyngeal Carcinoma: A 1:2 Matched Case–Control Study
Source: PLoS One. 2016 Oct 19;11(10):e0165131. doi: 10.1371/journal.pone.0165131 (PMC5070777; doi:10.1371/journal.pone.0165131)

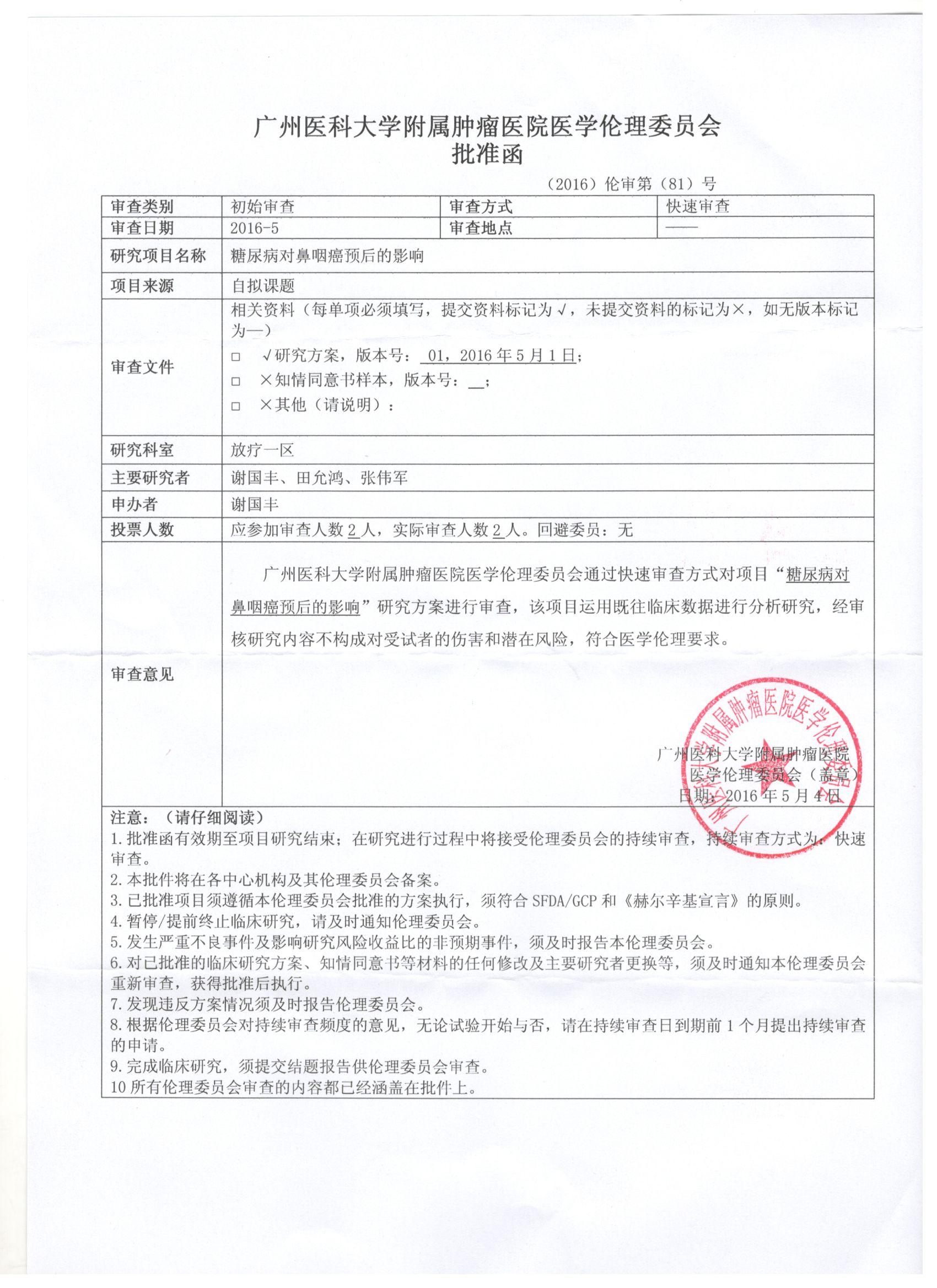

Supplement: S2 File — (ZIP) [file pone.0165131.s002.zip › Σ╝aτÉåμë╣Σ╗╢.jpg]
